# The presence of spin is commonly found in the abstracts of systematic reviews and meta‐analysis on robotic‐assisted unicompartmental knee arthroplasty

**DOI:** 10.1002/ksa.70014

**Published:** 2025-09-09

**Authors:** James Abesteh, Hassaan Abdel Khalik, Ayomide M. Ade‐Conde, Vickas Khanna, Etienne L. Belzile, Olufemi R. Ayeni

**Affiliations:** ^1^ Michael G. DeGroote School of Medicine McMaster University Hamilton Ontario Canada; ^2^ Division of Orthopaedic Surgery McMaster University Hamilton Ontario Canada; ^3^ School of Medicine Royal College of Surgeons in Ireland Dublin Ireland; ^4^ Division of Orthopaedic Surgery CHU de Québec‐Université Laval Québec City Québec Canada

**Keywords:** meta‐analysis, robotic‐assisted, spin, systematic review, unicompartmental knee arthroplasty

## Abstract

**Purpose:**

As robotic‐assisted unicompartmental knee arthroplasty (RA‐UKA) gains popularity, debate continues over its superiority to conventional UKA (C‐UKA). Systematic reviews and meta‐analyses (SRMAs) have examined this, but concerns exist about spin bias in their abstracts, which can significantly alter perceptions of a treatment's efficacy and safety. This study aims to evaluate the presence of spin bias in the abstracts of SRMAs comparing RA‐UKA and C‐UKA, and to assess the methodological quality of all included SRMAs using the AMSTAR‐2 tool.

**Methods:**

MEDLINE, EMBASE and the Cochrane Database of Systematic Reviews were searched from inception to 7 February 2025 for SRMAs that assessed RA‐UKA. Eligible studies assessed at least one outcome of RA‐UKA. Included studies were evaluated for the presence of spin in their abstracts using the methods outlined by Yavchitz et al. All full texts were subsequently assessed for methodological quality using the AMSTAR‐2 tool.

**Results:**

At least one element of abstract spin was identified in 12 of 16 included studies (75%). The most common category of spin was ‘misleading reporting’ in the form of *selective reporting of or overemphasis on efficacy outcomes or analysis favouring the beneficial effect of the experimental intervention*, observed in ten studies (63%). With the exception of one study with an overall ‘low’ confidence rating, all remaining studies were of ‘critically low’ A MeaSurement Tool to Assess systematic Reviews 2 (AMSTAR‐2) confidence. No study characteristics were significantly associated with the presence of abstract spin.

**Conclusion:**

The majority of RA‐UKA SRMAs contained spin, most commonly in the form of misleading reporting and interpretation, and were rated ‘critically low’ in quality by AMSTAR‐2. In a growing field like RA‐UKA where clinical decision‐making is influenced by SRMA results and conclusions, clinicians should critically review full texts to minimise the impact these biases may have on their practice.

**Level of Evidence:**

Level IV.

**Abbreviations**
ACL: anterior cruciate ligament
AMSTAR 2: A MeaSurement Tool to Assess systematic Reviews 2
CDSR: cochrane database of systematic reviews
CIs: confidence intervals
C‐UKA: conventional UKA
IQR: interquartile range
LOE: level of evidence
ORs: odds ratios
PRISMA: Preferred Reporting Items for Systematic Reviews and Meta‐analysis
RA‐TKA: robotic‐assisted total knee arthroplasty
RA‐UKA: robotic‐assisted unicompartmental knee arthroplasty
SD: standard deviation
SDC: supplemental digital content
SRMAs: systematic reviews and meta‐analyses
UKA: unicompartmental knee arthroplasty

## INTRODUCTION

Unicompartmental knee arthroplasty (UKA) is a well‐established treatment for end‐stage single‐compartment knee osteoarthritis, providing joint‐preserving benefits as a minimally invasive alternative to total knee arthroplasty [38, 41]. Although the overall utilisation of UKA has declined since 2021, the percentage of robotic‐assisted‐UKA (RA‐UKA) procedures has increased [14]. Recently, RA‐UKA has garnered significant attention due to its potential to further enhance surgical precision, optimise implant positioning and improve functional outcomes compared to conventional UKA (C‐UKA) [6, 15, 18, 33]. Despite the growing popularity of RA‐UKA, there remains ongoing debate about whether it can be clinically recommended as a superior intervention to C‐UKA [26, 37]. Systematic reviews and meta‐analyses (SRMAs) are the most comprehensive sources of evidence on this clinical question [55]. However, these studies are not immune to biases that can distort the interpretation of results and influence clinical decision‐making, especially when authors are eager to highlight the benefits of a novel intervention such as RA‐UKA.

As the body of literature on robotic‐assisted techniques grows, concerns have emerged about the potential for spin bias [10, 20, 28]. Spin bias refers to the selective or biased presentation of results that overstates the efficacy or safety of an intervention. As developed by Yavchitz et al., it can manifest in three main categories: *misleading representation*, *misleading reporting* and *inappropriate extrapolation* [56]. Since the abstracts of SRMAs are frequently the primary source of information guiding clinical decisions, the presence of spin in these sections can significantly alter perceptions of a treatment's efficacy and safety [4, 9]. In orthopaedics, spin has been evaluated in various contexts [21, 31, 43, 53, 54]. Hwang et al. found that spin is present in most SRMAs regarding the primary repair of the anterior cruciate ligament (ACL), often in the form of claiming safety or benefit for repair techniques while downplaying primary study methodologic weaknesses [31]. Further, Theismann et al. found a high prevalence of spin in abstracts investigating outcomes of hip arthroscopy for borderline hip dysplasia, such as an abstract claiming global improvement in patients with borderline hip dysplasia treated arthroscopically despite basing this claim on improvement in the surrogate marker of conversion rate to total hip arthroplasty [53]. However, despite its growing interest, no study has specifically evaluated spin bias in SRMAs comparing RA‐UKA and C‐UKA.

Using the PICO framework, we constructed the following research question: in SRMAs comparing RA‐UKA to C‐UKA, what is the prevalence of spin bias in abstracts and what is the methodological quality of the reviews? Thus, the primary aim of this study was to assess the presence and classification of spin bias in the abstracts of SRMAs comparing RA‐UKA and C‐UKA. The secondary aim was to evaluate the overall methodological quality of all included SRMAs using the AMSTAR‐2 critical appraisal tool [50]. By identifying the prevalence of spin bias and methodological limitations, this review aimed to provide insight into the credibility of existing evidence and its potential influence on adopting robotic‐assisted techniques over conventional methods.

## METHODS

This review was conducted following the Cochrane handbook guidelines and is reported in accordance with the Preferred Reporting Items for Systematic Reviews and Meta‐analysis (PRISMA) [29, 42].

MEDLINE, EMBASE and the Cochrane Database of Systematic Reviews (CDSR) were queried for SRMAs that assessed RA‐UKA using OVID. Searches were performed from database inception to 7 February 2025. The following search terms were used in the search strategy: ‘knee’, ‘arthroplasty’, ‘robotic’, ‘systematic review’ and ‘meta‐analysis’ (Supporting Information S1: Table S1).

The following inclusion criteria were used for this review: (1) systematic reviews or meta‐analyses comparing any outcome of RA‐UKA to C‐UKA, (2) human participants, (3) published in the English language. In order to maximise the inclusion of SRMAs, no restrictions on the level of evidence were placed. Exclusion criteria for this review were: (1) study only assessed RA‐UKA with no comparison to conventional technique, (2) study assessed both RA‐UKA and robotic‐assisted total knee arthroplasty (RA‐TKA) in combination.

### Study screening

The titles and abstracts of studies returned by the search strategy were independently screened in duplicate by two authors (H.A.K. and J.A.) according to the aforementioned eligibility criteria. Disagreements at this stage were moved on to the full‐text review to prevent premature exclusion. Full‐text screening was also conducted independently and in duplicate by the same two authors, with any disagreements at this stage resolved by consensus.

### Data abstraction from included studies

Two reviewers (J.A. and A.M.A.) abstracted data in duplicate from the included studies into an input form in Covidence (Veritas Health Innovation, Melbourne, Australia) which was designed *a priori*. The senior author (H.A.K.) was consulted to resolve any conflicts at the data abstraction stage. All abstracted characteristics and outcomes were prespecified. The following were abstracted from each study: (1) journal name and 2023 impact factor, (2) number of co‐authors, (3) number of included studies and knees, (4) funding source, (5) level of evidence, (6) outcomes reported and (7) country of the corresponding author as listed in the published article. The total number of citations to date was obtained from Goole Scholar on 8 March 2025, and citation density (the normalised number of citations per year published) was computed as a means to compare citations between papers published in different years. Google Scholar was selected for its inclusive coverage of journals. Level of evidence for each SRMA was determined based on the lowest level of evidence of the primary studies included, using the classification system published by the *Journal of Bone and Joint Surgery* [55].

### Assessment of spin bias and study quality

The nine critical domains outlined by Yavchitz et al. [56] were used to assess spin present in the abstracts of included SRMAs. The nine critical domains can be categorised as misleading reporting (*n* = 2), misleading interpretation (*n* = 5) and inappropriate extrapolation (*n* = 2) (Table 1). Assessment of spin in abstracts was done independently and in duplicate by two reviewers (J.A. and A.M.A.) with conflicts resolved by discussing with a senior author (H.A.K.). All included studies were eligible for spin assessment via this method given each SRMA contained an abstract.

**Table 1:** Types and categories of spin.

Spin category	Spin type
Misleading reporting	Type 3: Selective reporting of or overemphasis on efficacy outcomes or analysis favouring the beneficial effect of the experimental intervention
	Type 6: Selective reporting of or overemphasis on harm outcomes or analysis favouring the safety of the experimental intervention
Misleading interpretation	Type 1: Conclusion contains recommendations for clinical practice not supported by the findings
	Type 2: Title claims or suggests a beneficial effect of the experimental intervention not supported by the findings
	Type 4: Conclusion claims safety based on nonstatistically significant results with a wide confidence interval
	Type 5: Conclusion claims the beneficial effect of the experimental treatment despite high risk of bias in primary studies
	Type 9: Conclusion claims the beneficial effect of the experimental treatment despite reporting bias
Inappropriate extrapolation	Type 7: Conclusion extrapolates the review's findings to a different intervention (i.e., claiming efficacy of one specific intervention although the review covers a class of several interventions)
	Type 8: Conclusion extrapolates the review's findings from a surrogate marker or a specific outcome to the global improvement of the disease

*Note*: Classification of spin by Yavchitz et al. [56].

The AMSTAR 2 tool (A MeaSurement Tool to Assess systematic Reviews) was used to assess the quality of all included articles [50]. Briefly, the AMSTAR 2 tool uses 16 criteria to assess study quality, with seven of these criteria classified as ‘critical domains’. Studies are then assigned an overall level of confidence based on criteria fulfilled, with the authors of the tool advising against presenting percent scores [50]. Two reviewers (J.A. and A.M.A.) independently performed the quality assessment in duplicate with conflicts resolved by discussion with the senior author (H.A.K.). Both reviewers underwent a training phase of 10 studies to ensure accurate use of the AMSTAR‐2 tool. All included studies were eligible for this phase of analysis given the AMSTAR 2 tool is directly designed to be applied to SRMAs.

### Statistical analysis

After checking the normality of the data, the appropriate descriptive statistics, including medians and interquartile range (IQR) or weighted means and standard deviation (SD), were calculated for study characteristics. Categorical variables, including AMSTAR 2 criteria and spin bias domains, were reported as counts and percentages.

The primary outcome of this study was the presence or absence of spin in the included study abstracts. This was treated as a binary variable, with the presence of spin defined as an abstract having at least one category of spin. Additionally, we assessed the presence of spin against a number of prespecified study characteristics. Following previous studies that were unable to achieve adequate power for multivariable regressions [2, 21, 32], we computed the unadjusted odds ratios (ORs) and corresponding 95% confidence intervals (CIs) with these different study characteristics as the independent variables and the presence of spin as the dependent variable to explore possible correlations. Each of these study characteristics was considered a binary variable, with a study either meeting the characteristic (i.e., total citations ≥ 15) or not meeting the characteristic (i.e., total citations < 15). Thresholds for the binary outcomes were based on calculated medians and means, as appropriate. As in Reddy et al., odds ratios pertaining to spin and country of origin was also performed [45]. SPSS was used for statistical analysis. A *p*‐value < 0.05 was considered statistically significant.

## RESULTS

### Study characteristics and quality assessment

**Figure 1:**
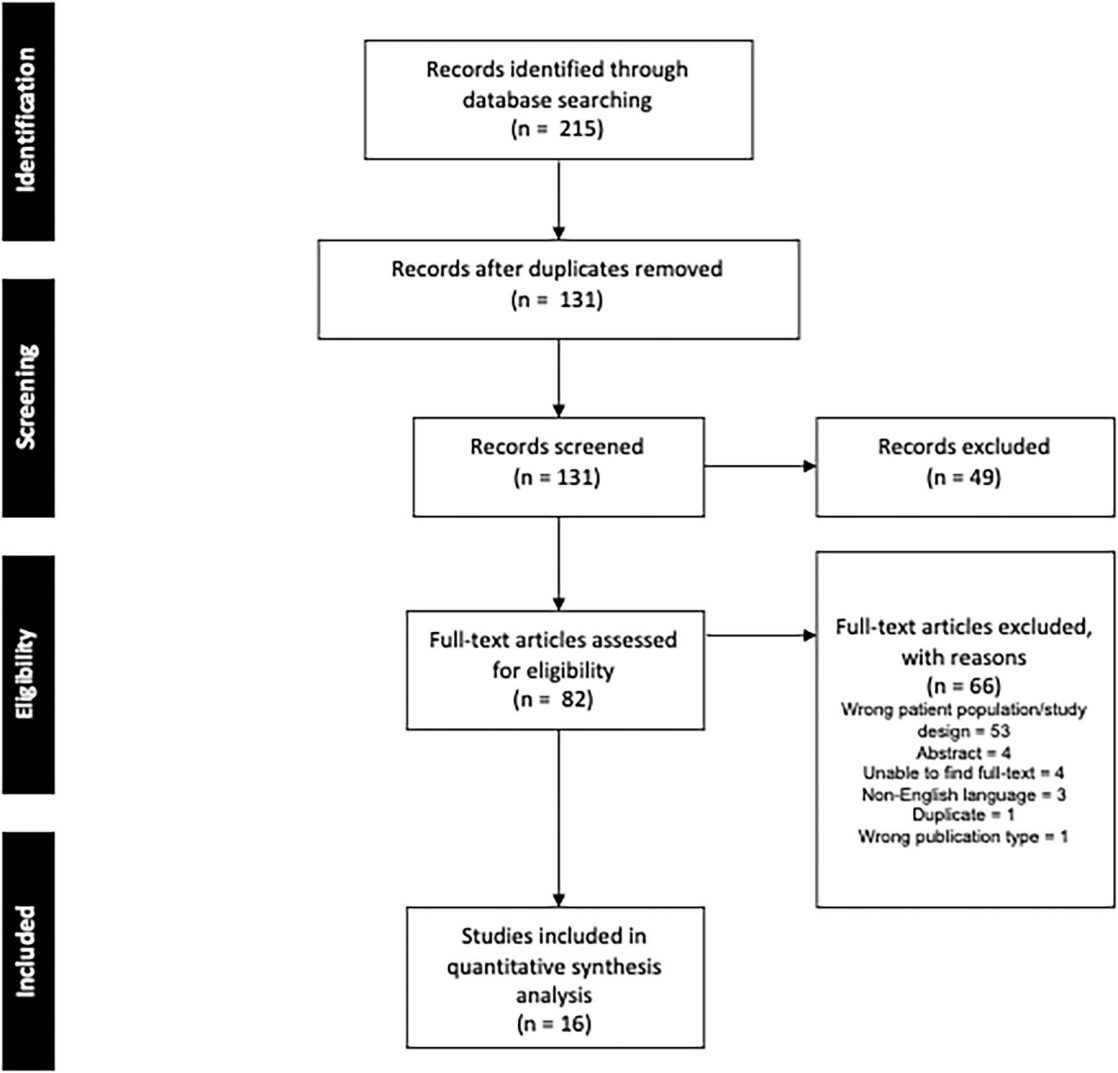
PRISMA flowchart.

The initial database search identified 215 studies, with 131 remaining for screening after the exclusion of duplicates (Figure 1). Sixteen studies were included in this review. Studies were published between 2018 to 2024 across 13 different journals (Table 2) and 16 different primary authors. Nearly one third of included studies (*n* = 5) were published from research groups based in China (Table 3). The median journal impact factor was 2.3 (interquartile range [IQR] 1.5–3.0) with a median of 15 citations (IQR 4–35). The median citation density was 3.6 (IQR 2–6). There was a mean of seven co‐authors (SD 2) across studies, with a median of 2176 knees (IQR 1087–14,499), across a median of 15 (IQR 9–19) includes studies per review.

**Table 2:** Included study characteristics.

Author (Year)	Journal name (IF)	Total citations (citation density)	Country	Authors, *n*	Study type	LOE	Number of studies, *n*	Number of knees, *n*	Spin present (Y/N)?
Are et al. (2023) [1]	Eur Rev Med Pharmacol Sci (3.3)	5 (2.5)	Italy	9	SR	IV	15	1262	N
Avram e al. (2024) [3]	J Pers Med (3)	0 (0)	Switzerland	10	SR and MA	III	19	2446	Y
Bensa et al. (2024) [7]	Bone Jt Open (2.8)	3 (3)	Switzerland	6	SR and MA	III	19	3074	Y
Bernard‐de‐Villeneuve e al. (2021) [8]	Arch Orthop Trauma Surg (2)	6 (1.5)	France	7	SR	III	9	50,100	N
Fu et al, (2018) [22]	Orthopade (1.004)	40 (5.7)	China	8	SR and MA	II	7	572	Y
Gaudiani et al. (2021) [24]	J Knee Surg (1.6)	32 (8)	United States	5	SR and MA	III	7	788	N
Ghazal et al. (2023) [25]	Cureus (1)	7 (3.5)	United Kingdom	6	SR and MA	III	16	1846	Y
Hoveidaei et al. (2024) [30]	Technol Health Care (1.4)	2 (2)	United States	8	SR and MA	III	5	1060	Y
Lin et al. (2020) [35]	Int J Med Robot (2.3)	30 (6)	China	4	SR	IV	21		Y
Mittal et al. (2021) [40]	Int J Med Robot (2.3)	4 (1)	South Korea	3	SR	III	53	20,087	N
Negrin et al. (2021) [44]	J Robot Surg (2.2)	18 (3.6)	Chile	8	SR	IV	15	2176	Y
Robinson et al. (2019) [46]	Bone Joint J (4.9)	69 (11.5)	United Kingdom	6	SR	IV	38	5793	Y
Sun et al. (2021) [52]	BMJ Open (2.4)	17 (4.3)	China	5	SR and MA	III	16	50,024	Y
Zhang et al. (2019) [57]	Medicine (Baltimore) (1.4)	36 (6)	China	6	SR and MA	II	11	1087	Y
Zhang et al. (2021) [59]	Int J Med Robot (2.3)	12 (2.4)	China	6	SR and MA	III	10	1231	Y
Zhang et al. (2022) [58]	Bone Joint J (4.9)	56 (18.7)	United Kingdom	8	SR and MA	IV	14	14,499	Y

Abbreviations: IF, impact factor; LOE, level of evidence; MA, meta‐analysis; SR, systematic review.

**Table 3:** Study characteristics.

Study characteristic	*N* (%)
Country	
Chile	1 (6.3)
China	5 (31.3)
France	1 (6.3)
Italy	1 (6.3)
South Korea	1 (6.3)
Switzerland	2 (12.5)
United Kingdom	3 (18.8)
United States	2 (12.5)
Study type	
SR	6 (37.5)
SR and MA	10 (62.5)
Funding	
Not funded	8 (50)
Public	4 (25)
Public and private	1 (6.3)
Not mentioned	3 (18.8)
LOE	
II	2 (12.5)
III	9 (56.3)
IV	5 (31.3)
Outcomes	
Patient reported outcomes	13 (81.3)
Objective outcomes (i.e., range of motion)	10 (62.5)
Complications/reoperations	14 (87.5)
Intraoperative outcomes (i.e., operative time, soft tissue injury)	9 (56.3)
Radiographic outcomes (includes implant positioning outcomes)	9 (56.3)
Resource utilisation (i.e., length of stay, cost)	5 (31.3)

*Note*: *N* = number of studies. Brackets denote percent of total included studies.

Abbreviations: MA, meta‐analysis; SR, systematic review.

Almost two‐thirds of included studies conducted some form of meta‐analysis (*n* = 10), with the majority of studies being LOE III (*n *= 9). Half of included SRMAs were not funded (*n* = 8), with another quarter receiving some form of public funding (*n* = 4). The most commonly reported outcomes were complications/reoperations (*n* = 14) and patient‐reported outcomes (*n* = 13).

### Assessment of spin

**Figure 2:**
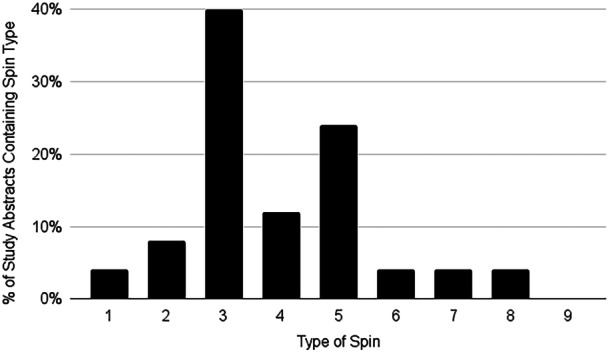
Frequency of spin types in included study abstracts.

At least one element of abstract spin was identified in 12 of 16 included studies (75%) [1, 3, 7, 8, 22, 25, 30, 35, 44, 46, 57, 58]. Of the identified instances of spin, almost half were related to misleading interpretation (48%), followed by misleading reporting (44%), and finally inappropriate extrapolation (8%). The most common form of spin was *selective reporting of or overemphasis on efficacy outcomes or analysis favouring the beneficial effect of the experimental intervention* (Type 3) which was present in 10 (63%) of studies (Table 4 and Figure 2). No study characteristics were found to have statistically significant odds ratios associated with the presence of abstract spin, but the power of this analysis was likely limited by the number of included studies (Table 5).

**Table 4:** Spin type prevalences.

Spin type (category of spin)	*N* (%)
(1) Conclusion contains recommendations for clinical practice not supported by the findings (misleading interpretation)	1 (6)
(2) Title claims or suggests a beneficial effect of the experimental intervention not supported by the findings (misleading interpretation)	2 (13)
(3) Selective reporting of or overemphasis on efficacy outcomes or analysis favouring the beneficial effect of the experimental intervention (misleading reporting)	10 (63)
(4) Conclusion claims safety based on nonstatistically significant results with a wide confidence interval (misleading interpretation)	3 (19)
(5) Conclusion claims the beneficial effect of the experimental treatment despite high risk of bias in primary studies (misleading interpretation)	6 (38)
(6) Selective reporting of or overemphasis on harm outcomes or analysis favouring the safety of the experimental intervention (misleading reporting)	1 (6)
(7) Conclusion extrapolates the review's findings to a different intervention (i.e., claiming efficacy of one specific intervention although the review covers a class of several interventions) (inappropriate extrapolation)	1 (6)
(8) Conclusion extrapolates the review's findings from a surrogate marker or a specific outcome to the global improvement of the disease (inappropriate extrapolation)	1 (6)
(9) Conclusion claims the beneficial effect of the experimental treatment despite reporting bias (misleading interpretation)	0 (0)

*Note*: *N* denotes the number of studies whose abstract contained each type of spin. Brackets denote percent of total included studies. This classification of spin is by Yavchitz et al. [56].

**Table 5:** Unadjusted odds ratios across presence of spin in abstract and various study characteristics.

Independent variable	Odds ratio	95% CI
Journal impact factor ≥2.3	0.33	0.03–4.19
Total citations ≥15	1.00	0.10–9.61
Citation density ≥3.6	1.00	0.10–9.61
Studies published from Asia	0.11	0.00–1.52
Number of co‐authors ≥7	0.56	0.31–1.00
Number of included studies ≥15	1.40	0.14–13.57
Number of included knees ≥2010	1.20	0.12–11.87

### Assessment of quality with AMSTAR‐2

Quality assessment using the AMSTAR‐2 tool demonstrated several areas of consistent methodologic limitations including a lack of explicit explanations for the selection of specific study designs (*n* = 16), missing lists of excluded studies with respective reasons for not including in the final review (*n* = 15) as well as a lack of further investigation of included studies' funding (*n* = 14) (Table 6). With the exception of one study with an overall ‘low’ confidence rating, all remaining studies were of ‘critically low’ confidence (Supporting Information S1: Table S2).

**Table 6:** AMSTAR quality assessment results.

Domain	Yes	Partial Yes	No
(1) Did the research questions and inclusion criteria for the review include the components of PICO?	15 (93.8)	‐	1 (6.3)
(2) Did the report of the review contain an explicit statement that the review methods were established prior to the conduct of the review and did the report justify any significant deviations from the protocol?	15 (93.8)	0 (0)	1 (6.3)
(3) Did the review authors explain their selection of the study designs for inclusion in the review?	0 (0)	‐	16 (100)
(4) Did the review authors use a comprehensive literature search strategy?	1 (6.3)	15 (93.8)	0 (0)
(5) Did the review authors perform study selection in duplicate?	11 (68.8)	‐	5 (31.3)
(6) Did the review authors perform data extraction in duplicate?	8 (50)	‐	8 (50)
(7) Did the review authors provide a list of excluded studies and justify the exclusions?	1 (6.3)	0 (0)	15 (93.8)
(8) Did the review authors describe the included studies in adequate detail?	3 (18.8)	8 (50)	5 (31.3)
(9) Did the review authors use a satisfactory technique for assessing the risk of bias (RoB) in individual studies that were included in the review?	6 (37.5)	6 (37.5)	4 (25)
(10) Did the review authors report on the sources of funding for the studies included in the review?	2 (12.5)	‐	14 (87.5)
a(11) If meta‐analysis was performed did the review authors use appropriate methods for statistical combination of results?	1 (10)	‐	9 (90)
a(12) If meta‐analysis was performed, did the review authors assess the potential impact of RoB in individual studies on the results of the meta‐analysis or other evidence synthesis?	1 (10)	‐	9 (90)
(13) Did the review authors account for RoB in individual studies when interpreting/discussing the results of the review?	3 (18.8)	‐	13 (81.3)
(14) Did the review authors provide a satisfactory explanation for, and discussion of, any heterogeneity observed in the results of the review?	7 (43.8)	‐	9 (56.3)
a(15) If they performed quantitative synthesis did the review authors carry out an adequate investigation of publication bias (small study bias) and discuss its likely impact on the results of the review?	3 (30)	‐	7 (70)
(16) Did the review authors report any potential sources of conflict of interest, including any funding they received for conducting the review?	12 (75)	‐	4 (25)

*Note*: ‘‐’ denotes response not applicable to domain. Data presented as *N* (%), where *N* is the number of studies classified as (yes/partial yes/no) for a given domain.

^a^ Denominator of studies adjusted to 10 as five studies did not perform meta‐analysis.

## DISCUSSION

RA‐UKA has risen in recent years, with reported benefits including increased accuracy, better functional outcomes, lower revision rates and higher patient satisfaction [14, 27, 34, 36, 49]. However, drawbacks like increased operating time, cost and limited intraoperative adaptability highlight the need for balanced reporting in SRMAs [5, 36]. Spin is a means of reporting an experimental treatment's results in a way that overstates the efficacy or safety of the treatment beyond that supported by a study's results, and surgeons should understand how spin can shape their views of the benefits and safety of RA‐UKA [56]. The primary finding of this review was that 75% of included studies contained at least one type of spin in their abstracts. Spin types can be categorised as either *misleading reporting*, *misleading interpretation* and *inappropriate extrapolation* [56]. The most common types of spin identified in abstracts were Type 3 (misleading reporting), Type 5 (misleading interpretation) and Type 4 (misleading interpretation), suggesting a relatively large prevalence of spin in RA‐UKA SRMA literature. All but one study was rated ‘critically low’ based on AMSTAR‐2 assessment. Univariate analysis did not show any statistically significant study characteristics associated with the presence of spin and was likely underpowered given the low number of included studies.

Previous studies on SRMA spin in other surgical disciplines reveals a wide range of spin prevalences such as 73% in plastic surgery and 10% in otolaryngology [23, 47]. Prior orthopaedic reviews have assessed the prevalence of spin in other areas of orthopaedics beyond RA‐UKA. One review of 96 studies assessed spin in abstracts of SRMAs of the surgical management of knee osteoarthritis, finding an overall spin prevalence of 35.4% with Type 3 spin being the most common [51]. A limitation noted by the authors was the need to investigate the prevalence of spin in specific surgical interventions. Our study shows the prevalence of spin in RA‐UKA SRMA abstracts specifically is twice as large at 75%—however, we similarly found Type 3 spin the most common type of spin. Another study found the spin prevalence in the treatment of proximal humerus fractures to be 34.2%—once again, this study also found Type 3 spin the most common [32]. The treatment of proximal humerus fractures is a more established topic compared to RA‐UKA, and the higher overall rate of spin in RA‐UKA studies may be in part due to optimism bias. Optimism bias, which has been previously documented in surgical research, occurs when the investment of surgeon time and resources into a new procedure influences them to believe it offers a benefit while simultaneously causing them to downplay its drawbacks [39]. While this may explain the high prevalence of Type 3 spin in our findings, other types of spin have different inherent sources. For example, Type 5 bias occurs when conclusions are based on primary studies with a high risk of bias [56]. Given this was the second most prevalent type of bias in our findings, a lack of high‐quality studies in RA‐UKA literature could be contributing to the high prevalence of spin. This is reflected in the LOE of included studies, with no studies above level II evidence. Additionally, only 37.5% of SRMAs adequately addressed AMSTAR‐2 item 9, which evaluates adequate risk of bias assessment. This suggests Type 5 spin might frequently be unintentional, as authors may not be aware their results are based on high‐risk studies if the risk of bias assessment is inadequate. Accordingly, we suggest that readers of RA‐UKA literature take time to review the LOE of included studies in reviews and familiarise themselves with common risk of bias assessment tools so they can make an informed decision on whether a proper risk of bias assessment was performed. Lastly, univariate analysis showed a correlation between absence of spin and higher journal impact factor. While not statistically significant given its wide confidence interval, this result might suggest readers should keep in mind that higher impact journals may have factors which could lead to a reduced incidence of spin in their publications, such as a propensity to attract studies with better methodology. Overall, the practical implication of the high prevalence in RA‐UKA SRMA abstracts is that readers should use a review's full‐text to come to fully‐informed conclusions rather than rely on the information presented in the abstract.

Of the types of spin, Type 3 was the most prevalent (63%), followed by Type 5 (38%) and Type 4 (19%). Type 3 spin occurs when an author selectively reports or overemphasises outcomes that favour the experimental group. For example, one study's abstract results reported several benefits of MAKO‐assisted UKA over conventional UKA but failed to mention a statistically significant longer operating time for RA‐UKA [35]. Similar methodological orthopaedic studies with a large number of included reviews have found a prevalence of Type 3 spin between 15.6% and 53.5%, Type 5 spin between 5.2% and 16.3%, and Type 4 spin between 0% and 25% [13, 32, 51]. Given spin in abstracts has been shown to increase a reader's interest in the full‐text and lead to an experimental treatment as being viewed as more beneficial, readers of literature in developing fields such as RA‐UKA should carefully evaluate claims in abstracts against full‐text results/conclusions and ensure they are exposing themselves to a balanced selection of articles on a given topic [12]. Additionally, readers who review RA‐UKA literature through platforms that primarily present abstracts and not full‐text articles should instead use these platforms as a starting point for browsing RA‐UKA rather than as final sources on the topic.

Based on prior studies assessing the quality of SRMAs in orthopaedic surgery using the AMSTAR‐2 tool, confidence in included studies has been found to be ‘critically low’ in 83% of studies and up to 93% in the spine literature [17, 31, 48]. One strategy to improve AMSTAR‐2 scores in RA‐UKA and other orthopaedic research is to increase awareness of the AMSTAR‐2 system. A study assessing barriers to AMSTAR‐2 adoption found that among authors who had used the AMSTAR criteria but not the AMSTAR‐2 criteria, 23% were not aware AMSTAR‐2 was published [11]. Increasing awareness of the criteria might also improve AMSTAR‐2 ratings by enabling authors to proactively incorporate criteria elements into study design, such as reporting excluded studies and their criteria for exclusion and sources of included study funding (two criteria often absent in studies we assessed). Another recommendation is that authors should carefully assess included studies for funding sources. Not only was this a commonly missed AMSTAR 2 criteria, but conflicts of interest in orthopaedic research are known to be associated with positive outcomes and a recent study on financial conflicts of interest in robotic arthroplasty found a majority of studies (91%) contained a relevant author disclosure [16, 19]. Furthermore, assessing included articles for sources of funding provides additional discussion points for authors to comment on when describing the impact of risk of bias on meta‐analysis review results, two additional domains often neglected by authors in our findings.

Our study has several limitations. The inherent degree of subjectively of spin assessment can contribute to result variability, and each author's own experiences in clinical practice and education in research methods will inevitably contribute to this—we attempted to minimise this by independent, duplicate assessment of article spin. Additionally, our study limited spin assessment to abstracts, and it is not known to what degree spin in RA‐UKA abstracts might predict spin the full‐text. Our study was also unable to achieve adequate power for a multivariable regression. Finally, a limited number of studies and resultant statistical constraints rendered it challenging to proceed with additional analyses across other publication characteristics such author disclosures. Despite this, our study provides a comprehensive assessment of spin bias in RA‐UKA SRMAs and reveals areas for future studies to improve on. Together, these findings highlight the need for a greater awareness of spin and more balanced reporting of study findings in future RA‐UKA investigations to ensure reliable, high‐quality evidence guides the future of the field.

## CONCLUSION

The majority of RA‐UKA SRMAs contained spin, most commonly in the form of misleading reporting and interpretation, and were rated ‘critically low’ in quality by AMSTAR‐2. The presence of spin in abstracts can potentially mislead readers, particularly those relying solely on abstracts to judge a study's key findings. In a growing field like RA‐UKA where clinical decision‐making is influenced by SRMA results and conclusions, clinicians should critically review full texts to minimise the impact these biases may have on their practice.

## AUTHOR CONTRIBUTIONS

Hassaan Abdel Khalik was involved in the study conception, study screening, data abstraction, data analysis and manuscript writing. James Abesteh was involved in the study screening, data abstraction and manuscript writing. Ayomide M. Ade‐Conde was involved in the data abstractions and manuscript writing. Etienne L. Belzile, Olufemi R. Ayeni and Vickas Khanna were involved in manuscript writing and review.

## CONFLICT OF INTEREST STATEMENT

Dr. Vickas Khanna reports consulting fees from Stryker, Bioventus and Zimmer Biomet; honorarium from Sanofi; travel support from Ossur; and that his spouse is employed by Stryker. Dr. Ayeni reports consulting fees from Stryker, serves as President of the Canadian Orthopaedic Association, is the owner of Notch Academy, and holds a Tier 2 Canada Research Chair. Dr. Belzile reports grants from CIHR, MITACS, AOSSM, the Arthritis Society, and Canadian Blood Services; royalties from BodyCad; honorarium from PendoPharm; and serves on the COA Continued Education Committee and as an Associate Editor for OTSR. The remaining authors have no disclosures to declare.

## ETHICS STATEMENT

The authors have nothing to report.

## Supporting information

Supplemental Digital Content Table 1 and Table 2.

## Data Availability

The data that support the findings of this study are available from the corresponding author upon reasonable request.
