# Supplementary material for: The presence of spin is commonly found in the abstracts of systematic reviews and meta‐analysis on robotic‐assisted unicompartmental knee arthroplasty
Source: Knee Surg Sports Traumatol Arthrosc. 2025 Sep 9;34(2):574–85. doi: 10.1002/ksa.70014 (PMC12850591; doi:10.1002/ksa.70014)
Supplement: Supplementary file 1 — Supplemental Digital Content Table 1 and Table 2. [file KSA-34-574-s001.docx]

**Supplemental Digital Content Table 1** Search Strategy

| MEDLINE, EMBASE and Cochrane Database of Systematic Reviews via OVID |
| --- |
| 1. exp Knee/  2. exp Knee Joint/  3. knee.ti,ab.  4. exp Arthroplasty/  5. exp Arthroplasty, Replacement/  6. exp Arthroplasty, Replacement, Knee/  7. arthroplasty.ti,ab.  8. replacement.ti,ab.  9. tka.ti,ab.  10. tkr.ti,ab.  11. uka.ti,ab.  12. unicompartmental.ti,ab.  13.exp Joint Prosthesis/  14. prosthes*.ti,ab.  15. exp Robotic Surgical Procedures/  16. robot*.ti,ab.  17. robotic-assisted.ti,ab.  18. robotic arm-assisted.ti,ab.  19. robot-assisted.ti,ab.  20. robotic-arm.ti,ab.  21. robotic.ti,ab.  22. robot.ti,ab.  23. systematic review.ti,ab.  24. systematic-review.ti,ab.  25. meta analysis.ti,ab.  26. meta-analysis.ti,ab.  27. exp "Systematic Review"/  28. exp Meta-Analysis/  29. 1 or 2 or 3  30. 4 or 5 or 6 or 7 or 8 or 9 or 10 or 11 or 12 or 13 or 14  31. 15 or 16 or 17 or 18 or 19 or 20 or 21 or 22  32. 23 or 24 or 25 or 26 or 27 or 28  33. 29 and 30 and 31 and 32 |

**Supplemental Digital Content Table 2** Study-specific quality assessment

| Study | 1 | 2 | 3 | 4 | 5 | 6 | 7 | 8 | 9 | 10 | 11 | 12 | 13 | 14 | 15 | 16 | Critical | Non-Critical | Overall |
| --- | --- | --- | --- | --- | --- | --- | --- | --- | --- | --- | --- | --- | --- | --- | --- | --- | --- | --- | --- |
| Are et al (2023) [1] |  |  |  |  |  |  |  |  |  |  |  |  |  |  |  |  | 2 | 5 | CL |
| Avram e al (2024) [3] |  |  |  |  |  |  |  |  |  |  |  |  |  |  |  |  | 5 | 5 | CL |
| Bensa et al (2024) [7] |  |  |  |  |  |  |  |  |  |  |  |  |  |  |  |  | 3 | 3 | CL |
| Bernard-de-Villeneuve e al (2021) [8] |  |  |  |  |  |  |  |  |  |  |  |  |  |  |  |  | 2 | 3 | CL |
| Fu et al (2018) [22] |  |  |  |  |  |  |  |  |  |  |  |  |  |  |  |  | 4 | 7 | CL |
| Gaudiani et al (2021) [24] |  |  |  |  |  |  |  |  |  |  |  |  |  |  |  |  | 3 | 7 | CL |
| Ghazal et al (2023) [25] |  |  |  |  |  |  |  |  |  |  |  |  |  |  |  |  | 4 | 4 | CL |
| Hoveidaei et al (2024) [30] |  |  |  |  |  |  |  |  |  |  |  |  |  |  |  |  | 4 | 4 | CL |
| Lin et al (2020) [35] |  |  |  |  |  |  |  |  |  |  |  |  |  |  |  |  | 4 | 6 | CL |
| Mittal et al (2021) [40] |  |  |  |  |  |  |  |  |  |  |  |  |  |  |  |  | 3 | 5 | CL |
| Negrin et al (2021) [44] |  |  |  |  |  |  |  |  |  |  |  |  |  |  |  |  | 2 | 4 | CL |
| Robinson et al (2019) [46] |  |  |  |  |  |  |  |  |  |  |  |  |  |  |  |  | 1 | 4 | L |
| Sun et al (2021) [52] |  |  |  |  |  |  |  |  |  |  |  |  |  |  |  |  | 3 | 3 | CL |
| Zhang et al (2019) [57] |  |  |  |  |  |  |  |  |  |  |  |  |  |  |  |  | 2 | 3 | CL |
| Zhang et al (2021) [59] |  |  |  |  |  |  |  |  |  |  |  |  |  |  |  |  | 4 | 4 | CL |
| Zhang et al (2022) [58] |  |  |  |  |  |  |  |  |  |  |  |  |  |  |  |  | 3 | 4 | CL |

CL, critically low; L, low
